# Multivariate Statistical Analysis as a Supplementary Tool for Interpretation of Variations in Salivary Cortisol Level in Women with Major Depressive Disorder

**DOI:** 10.1155/2015/987435

**Published:** 2015-08-25

**Authors:** Ewelina Dziurkowska, Marek Wesolowski

**Affiliations:** Department of Analytical Chemistry, Medical University of Gdansk, Gen. J. Hallera 107, 80-416 Gdansk, Poland

## Abstract

Multivariate statistical analysis is widely used in medical studies as a profitable tool facilitating diagnosis of some diseases, for instance, cancer, allergy, pneumonia, or Alzheimer's and psychiatric diseases. Taking this in consideration, the aim of this study was to use two multivariate techniques, hierarchical cluster analysis (HCA) and principal component analysis (PCA), to disclose the relationship between the drugs used in the therapy of major depressive disorder and the salivary cortisol level and the period of hospitalization. The cortisol contents in saliva of depressed women were quantified by HPLC with UV detection day-to-day during the whole period of hospitalization. A data set with 16 variables (e.g., the patients' age, multiplicity and period of hospitalization, initial and final cortisol level, highest and lowest hormone level, mean contents, and medians) characterizing 97 subjects was used for HCA and PCA calculations. Multivariate statistical analysis reveals that various groups of antidepressants affect at the varying degree the salivary cortisol level. The SSRIs, SNRIs, and the polypragmasy reduce most effectively the hormone secretion. Thus, both unsupervised pattern recognition methods, HCA and PCA, can be used as complementary tools for interpretation of the results obtained by laboratory diagnostic methods.

## 1. Introduction

An efficient and accurate diagnosis is of primary importance for clinical care. A wide range of laboratory diagnostic methods has been developed to support strategies of disease control. Proper evaluation of large matrices of the data acquired with the aid of modern laboratory diagnostic techniques involves the use of advanced statistical methods. Multivariate statistical analysis is one that seems to be very useful to solve that problem. They enable us to explain the meaning of the multidimensional data in the mathematic and statistic way and to enable extraction of the most useful information from the complicated data sets.

Multivariate statistics includes both linear and nonlinear statistical tools that can be used in order to understand the relationships between variables and their relevance to the problem being studied [1–4]. There are many different multivariate models, each with its own type of analysis, for instance, multivariate analysis of variance (MANOVA), principal component analysis (PCA), discrimination analysis (DA), partial least squares (PLS) and their variants, cluster analysis (CA), and various types of artificial neural networks. These methods are very helpful in bioprocess data analysis [1, 4].

In multivariate statistics a data matrix is created in two dimensions, where the samples in the rows are described by variables in columns [2, 3]. PCA enables reduction of the number of possibly correlated variables into the smaller value of orthogonal ones. It is one of the most popular multivariate data analysis tools that can be applied to find correlation between variables and to observe changes in them. PLS allows us to find the latent relations between variables and is useful as a discrimination tool. In that way PLS is similar to PCA, while CA enables us to measure the similarities and dissimilarities between the samples and to classify them into groups. In that way CA simply discovers structures in the multidimensional data without explaining why they exist.

PCA is widely used in medical studies. As claimed in the literature, this method has been used as supplementary tool facilitating diagnosis of some diseases, for instance, cancer [5–8], allergy [9, 10], pneumonia [11], or Alzheimer's disease [12]. The study of changes in cancer tissues by inspection of relationships between levels of trace elements (Pb, Al, Zn, Cd, Cu, Ni, and Co) in laryngeal cancer and healthy tissues suggests that PCA can differentiate the cancer and healthy tissues [7]. This method was also used to expose differences in the levels of various essential elements in serum and arterial wall of patients with atherosclerosis obliterans and the control group [13] and to discriminate between the levels of metabolites, such as amino acids and alcohols in serum of patients with oral cancer and healthy subjects [8]. Moreover, PCA was found to be an effective tool for grouping patients with the fourth stage of breast cancer and healthy ones into separate clusters based on the blood levels of hydroxylated phospholine lipids [6]. For better interpretation of the data, PCA is frequently combined with CA. This combination was used as a supplementary tool for diagnosis of Alzheimer's disease based on the serum concentration of multivalent cations [12]. The results show that both techniques can be useful for early detection of Alzheimer's disease enabling efficient therapy.

Multivariate statistics is also applied in psychiatry for solving problems due to interpretation of the data acquired for patients with major depressive disorder (MDD) [14] and bipolar disorder (BP) [15]. PLS has shown that the proton nuclear magnetic resonance (NMR) spectra of blood plasma of the depressed patients differ significantly from those of the control group [14]. Thus, NMR spectroscopy could be considered as a useful tool for the diagnosis of depression. The NMR spectroscopy was also used to study the blood serum metabolic profiles of patients with BP under different treatments [15]. Taking into account the levels of lipids, lipoproteins, and amino acids in blood serum of these patients, PCA and PLS suggest that the changes in metabolic profile of blood serum can be associated with the treatment. Gas chromatography/mass spectrometry coupled with multivariate data analysis tools has shown that the metabolic profiles of blood plasma can also be used as a novel laboratory-based test for diagnosis MDD and its subtypes (early life stress/MDD and nonearly life stress/MDD) [16]. Furthermore, hierarchical cluster analysis (HCA) was found to be a profitable tool for classifying personality profiles in women with perinatal depression [17].

The above literature screening shows that multivariate statistical analysis is a beneficial tool in the medical sciences for solving the complex relations between objects and variables in the multivariate databases. Therefore, the aim of this study was to use two unsupervised pattern recognition techniques, hierarchical cluster analysis (HCA) and principal component analysis (PCA), to seek the relationship between the antidepressants used in the therapy and the cortisol level and hospitalization periods of subjects with major depressive disorder (MDD). For this reason, the levels of the hormone were determined in saliva obtained from depressed women during their hospitalization, and the acquired matrix of the data was examined by advanced multivariate statistical methods, HCA and PCA.

## 2. Experimental Part

### 2.1. Participants

Women with MDD defined according to the International Classification of Diseases (ICD-10) were recruited into the study at the Hospital for Nervous and Mental Diseases in Starogard Gdanski (Poland). The enrolment was based on the clinical interview with psychiatrist. The subject was informed about the aim of the study and was asked for their written consent to participate in the study. They were also informed that they can refrain from participating in the study at any time if desired. The participants were excluded if they did not understand the meaning of the study or when participating in the study could be detrimental to their well-being. Pregnancy and breastfeeding were also excluding factors. Finally, 97 women with MDD were included in this study. The mean age of the participants was 48 (±10) years and mean period of hospitalization was 42 (±24) days. Multiplicity of hospitalization was 3 (±2) times. The study had been approved by the ethical committee of the Medical University of Gdansk, Poland.

### 2.2. Materials

Saliva obtained from depressed women treated with different antidepressants was used in this study. Because the hormone is secreted in the diurnal cycle and its highest level occurs in the morning, the samples were collected without any stimulation into plastic tube, every day about 10 a.m., during the whole period of hospitalization. The subjects were instructed to rinse the mouth with water and not to eat or drink about half an hour before the collection. After collection saliva samples were transported to the Medical University of Gdansk, where they were frozen until the analysis.

### 2.3. Hormone Assay

To quantify the salivary cortisol a HPLC procedure with UV detection was developed [18]. A mixture of acetonitrile and water (30 : 70; v/v) was taken as a mobile phase and a chromatographic column with C_18_ packing was a stationary phase. For calibration an internal standard, carbamazepine, was applied. The hormone was isolated from saliva by liquid-liquid extraction with dichloromethane.

### 2.4. Statistical Methods

All statistical calculations were carried out using Statistica 10 (StatSoft, Cracow, Poland) software. The level of statistical significance was set at *p* < 0.05. The Wilcoxon test was used for assessment of impact of the antidepressant therapy on the mean cortisol level during three periods of hospitalization. This test is an equivalent to Student's* t*-test. As a nonparametric statistical pattern it can be used for comparing two sets of samples or repeated measurements on a single sample. ANOVA test (one-way analysis of variance) was applied for evaluation of the impact of antidepressants on the hospitalization period as well as the mean and final levels of cortisol. This test is used for comparing the mean values of three or more sets of samples. Moreover, for assessment of statistically significant differences among four HCA clusters, ANOVA test with the NIR test as a* post hoc* analysis was used.

To establish a relationship between the antidepressants as well as the cortisol level and hospitalization period due to MDD, HCA and PCA were used. For both multivariate techniques, a matrix with 16 variables characterizing 97 patients was created. The matrix included the patients' age, multiplicity and period of hospitalization, initial and final cortisol levels, its highest and lowest concentrations, and also the difference between them. Furthermore, mean concentrations and medians determined during the whole period of hospitalization as well as the mean levels of hormone in different hospitalization phases were also used. The best results were obtained using Ward's hierarchical agglomeration with Euclidean distance measure in HCA and strategy without the rotation of factors in PCA.

## 3. Results

97 patients participated in this study who are hospitalized at the Hospital for Nervous and Mental Diseases in Starogard Gdanski (Poland). About 2700 saliva samples were collected from patients into plastic tube every morning during the whole period of hospitalization. The mean age of the patients was 48 years and the mean period of hospitalization was 41 days. As shown in Table 1, for the treatment of depression, antidepressants with different mechanism of action and defined daily dosage were used during the whole period of hospitalization. In some cases either combination treatment or neuroleptics, like olanzapine or perazine, were applied.

ANOVA test demonstrates that antidepressants used for the treatment did not have a significant impact on the hospitalization period (*p* = 0.1160, *F* = 1.6416). Moreover, this test also shows that the therapy has no influence on either the mean salivary level of the hormone (*p* = 0.6263, *F* = 0.7899) or the final cortisol level (*p* = 0.8190, *F* = 0.5690). The Wilcoxon test was carried out to indicate statistical differences among the mean cortisol levels in different hospitalization periods. This test has shown that there is a statistical difference between the mean concentrations in 30% and 60% of the hospitalization periods. The most significant differences were found during the treatment with TCAs (tricyclic antidepressants) (*p* = 0.0229, *Z* = 2.2749), SSRIs (selective serotonin reuptake inhibitors) (*p* = 0.0003, *Z* = 3.6434), SNRIs (serotonin and norepinephrine reuptake inhibitors) (0.0008, *Z* = 3.3510), SSAs (specific serotonin antidepressants) (*p* = 0.0280, *Z* = 2.1974), polypragmasy (*p* = 0.0004, *Z* = 3.5162), and neuroleptics (*p* = 0.0058, *Z* = 2.7562). However, there were no differences between the mean cortisol levels in 60% and 90% of hospitalization periods. In the case of the mean levels of cortisol in 30% and 90% of the hospitalization period, there were the differences when the patients were treated with SSRIs (*p* = 0.0031, *Z* = 2.9603), SNRIs (*p* = 0.0012, *Z* = 3.2374), polypragmasy (*p* = 0.0006, *Z* = 3.4128), and neuroleptics (*p* = 0.0044, *Z* = 2.8451).

**Figure 1:**
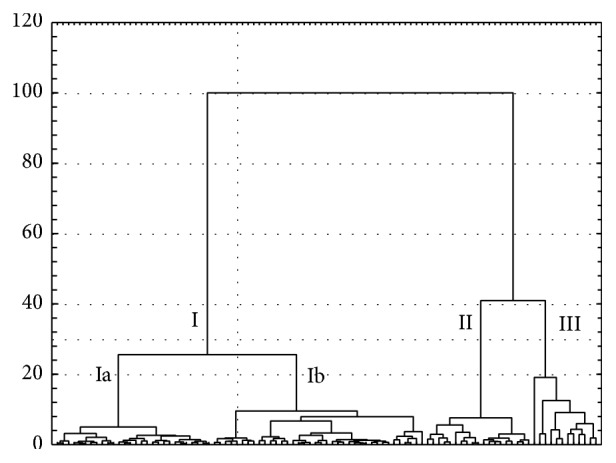
HCA dendrogram illustrating the clustering of ninety seven patients under antidepressant therapy.

The data set acquired in this study was subjected to hierarchical cluster analysis (HCA) and principal component analysis (PCA) to establish the relationships among subjects under antidepressant therapy with different active pharmaceutical ingredients. The results of HCA are presented in Figure 1. There are three clusters at a level of 1/3 of the maximum distance. The majority of the patients are grouped in cluster I, which is divided into two subclusters (Ia and Ib) at the level of 1/4 of the maximum distance. Patients with the low mean cortisol concentration when the highest hormone level was lower than 31 ng/mL are grouped in cluster Ia. Furthermore, in all cases the final cortisol concentrations were lower than 10 ng/mL. SSRIs and TCAs are the most commonly used drugs in the antidepressant therapy.

Cluster Ib is formed by subjects with the mean cortisol concentrations between 3 and 24 ng/mL. Also the mean level of cortisol in different periods of hospitalization was higher and was in the range from 1 to 45 ng/mL. In some cases the final cortisol concentration was above the reference value, and the highest one amounted to 42 ng/mL. In this cluster 11 patients were treated with combination therapy mainly with SSRIs and SNRIs or SSAs.

Clusters II and III are joined with cluster I at the maximum distance. Cluster II is created by patients with the mean cortisol level higher than the reference value. The level of the hormone was in the range between 10 and 31 ng/mL. Also the final concentration was higher (mostly a dozen or so ng/mL), but in some cases it was the several dozen ng/mL. The mean level of hormone determined in the different periods was between 4 and 83 ng/mL. In this group only SSRIs and SNRIs antidepressants were applied. There were no neuroleptics and the polypragmasy was used only in three cases. The majority of patients who formed cluster II were hospitalized between 29 and 82 days.

The last cluster is formed by patients with very high final and mean levels of the hormone determined during the 30%, 60%, and 90% of the hospitalization period. In all the cases the hospitalization was longer than 29 days.

The selected characteristic features of the patients created four clusters in Figure 1 are compiled in Table 2. The ANOVA test shows that the patients' age, multiplicity of hospitalization, lowest cortisol concentration, and median determined during the whole period of hospitalization did not have a significant impact on grouping the subjects into four clusters. However, the statistically significant differences between these clusters were found in the case of the highest cortisol concentration and the difference between highest and lowest cortisol concentration as well as standard deviation and relative standard deviation of mean cortisol concentration. This test also showed that there is a statistical difference between cluster III and remaining clusters taking into account the final cortisol level and mean level of hormone during the 90% of the hospitalization period.

**Figure 2:**
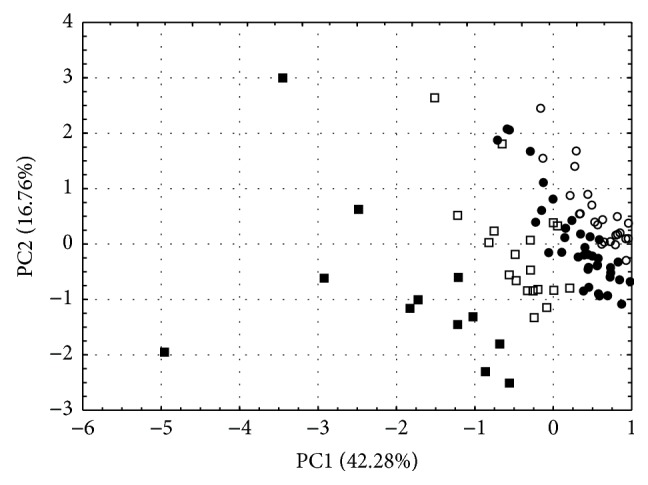
PCA scores plot illustrating the grouping of ninety-seven patients under antidepressant therapy. ○: cluster Ia, ●: cluster Ib, □: cluster II, and ■: cluster III.

The second multivariate approach, PCA, creates two first principal components (PC1 and PC2) that explain more than 59% of the data variability.  Figure 2 illustrates a PCA score plot in the form of a two-dimensional plane. It confirms the results obtained by HCA. In both cases, patients formed three groups. The first one is created by subjects with initial cortisol concentration lower than 40 ng/mL. In the majority of cases the hormone level falls in the range of a dozen or so ng/mL. Also the mean level was dozen of ng/mL and the highest one the most often is the initial one. On the other hand, patients with a very high initial cortisol level and at the same time the high mean level of the hormone in the first period of hospitalization (30%) are grouped in cluster III. The same women formed the third cluster in HCA (Figure 1).

**Figure 3:**
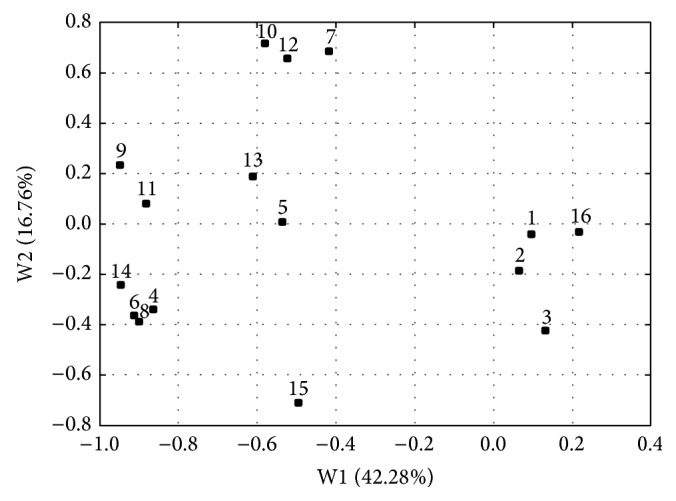
PCA loadings plot illustrating the impact of fourteen raw variables on the scattering of ninety-seven patients under antidepressant therapy. The Arabic digits denote the raw variables as follows: 1: patients age, 2: multiplicity of hospitalization, 3: period of hospitalization, 4: initial cortisol level, 5: final cortisol level, 6: the highest cortisol concentration, 7: the lowest cortisol concentration, 8: difference between the highest and lowest cortisol concentration, 9: mean concentration, 10: median determined during the whole period of hospitalization, 11: mean level of hormone during the 30% of the hospitalization period, 12: mean level of hormone during the 60% of the hospitalization period, 13: mean level of hormone during the 90% of the hospitalization period, 14: standard deviation of the mean concentration, 15: relative standard deviation of the mean concentration, and 16: antidepressant.

Figure 3 shows the PCA loadings, that is, the relationship between the raw variables and calculated principal components. The raw variables, which located the subjects according to the PC1 axis, were the mean, initial, and the highest salivary cortisol levels, the difference between highest and lowest cortisol concentration, the mean level of hormone during the 30% of hospitalization period, and the standard deviation of mean cortisol concentration. The most significant impact on the characteristic scattering of the subjects according to PC2 axis had the median, the lowest, and the mean levels of cortisol during the 60% of hospitalization as well as the relative standard deviation of mean cortisol concentration, which is negatively correlated with this axis.

## 4. Discussion

To disclose the relationship between the drugs used in the therapy of MDD and the salivary cortisol level as well as the period of hospitalization, 97 patients were treated with various groups of antidepressants. The largest group of the patients was treated with SSRIs that are the first-line drugs in the treatment of depression. These drugs have lower side effects in comparison with older TCAs. In this study 28 patients received SSRIs in monotherapy whereas 11 subjects were treated with SSRIs in polypragmasy. The second group of the most commonly used antidepressants was SNRIs. Venlafaxine, which was used by 14 patients in monotherapy, is only the one active pharmaceutical ingredient from this group that is applied in the therapy of depression in Poland. SNRIs are a new group of drug substances that act as inhibitors of serotonin and norepinephrine, and also by low increase in the dopamine concentration. The latter effect was found to be helpful in the treatment, especially for patient with decreased activity. Both patients with severe depression and patients of advanced age with any kind of depression are treated with TCAs. In this study 12 women were treated with tricyclic antidepressants in monotherapy, despite their numerous side effects [19].

Inspection of the data listed in Table 1 shows that the mean final level of cortisol was lower in almost all the therapies. Only in the case of paroxetine the mean initial hormone level was lower than the final one. Furthermore, the majority of therapies decrease the cortisol concentration to the reference values. As reported in the literature, the salivary cortisol level of a healthy person in the morning should fall within the concentration range between 1 and 8 ng/mL [20]. Moreover, antidepressants used in polypragmasy much more strongly affected cortisol secretion and in all cases the reduction in hormone concentration was observed.

ANOVA test indicates that any of treatments do not affect the hospitalization period or the mean cortisol concentration. However, the Wilcoxon test revealed that some of the therapies enabled a better control of the hormone secretion. Among the ten different therapies used for the treatment of depression, four of these were the most effective. The therapies with SSRIs, SNRIs, polypragmasy, and neuroleptics decrease the cortisol level in the first fraction of hospitalization (significant differences between 30% and 60% of the hospitalization period). At the same time there were no differences in the cortisol levels between 60% and 90% of hospitalization, when these groups of drugs were used. The fluctuation of cortisol secretion did not increase in the third period of hospitalization as demonstrated by significant differences between 30% and 90% of hospitalization and no statistical differences between the second and third one were found.

In the case of TCAs and SSAs, the Wilcoxon test did not show significant differences between the mean concentrations of cortisol quantified in the same hospitalization period. These results can be due to fluctuation of the hormone level. On the one hand, in the first fraction of hospitalization the cortisol secretion decreased and at the end of the treatment (about 30th day) its level increased and the mean concentration was elevated. On the other hand, the cortisol secretion was raised at the beginning by only a few ng/mL and in second and third fraction of cure the level fell to the referential values. The differences between the absolute values were of the order of a few ng/mL, but at the same time they were a few times higher. Examples of this type of cortisol secretion are patients treated with TCAs.

Statistically significant differences between four clusters of the patients are due to concentration of cortisol, especially the initial and highest one but also the difference between highest and lowest cortisol concentration. It is difficult to identify which class of the drugs has the strongest power to reduce the secretion of hormone, because in all clusters all types of drugs are included. That is why it can be stated that this is individual differences in response to treatment, though some trends exist. In the first cluster 25% of the patients were treated with SSRIs (above 50% of all treated with SSRI) and 19% with polypragmasy (more than 26% of all treated this way), 14% with TCAs and almost 13% with others psychoactive drugs (80% treated with neuroleptics). In this cluster the fluctuation of the cortisol concentration during the whole period of hospitalization was the lowest. Also there were no significant differences between subclusters Ia and Ib in mean level of the hormone and the mean concentration of cortisol in the 30% of hospitalization, but there were the differences between these subclusters and two remaining. Moreover, the mean concentration of cortisol in the 30% of hospitalization was different in this cluster than in clusters II and III.

To sum up, multivariate statistical analysis has shown that there are no explicit results demonstrating which of the antidepressants had the greatest impact on the hospitalization period. In some cases it can be stated that there is a tendency to grouping the patients based on the influence of the treatment on the cortisol secretion. Both multivariate techniques have shown that in the first cluster there are the majority of the patients treated with TCAs, SSRIs, SNRIs, polypragmasy, and neuroleptics. This group is characterized by a small fluctuation of the hormone secretion. The best results of decreasing the cortisol concentration were achieved in the case of SSRI and polypragmasy treatment. The substantial group of patients treated with these antidepressants is grouped in cluster Ia, where the fluctuation of cortisol secretion during the whole period of hospitalization is the lowest.

The results obtained by HCA and PCA were confirmed by Wilcoxon test, which revealed that antidepressants, such as TCAs, SSRIs, SNRIs, SSAs, or polypragmasy, but also neuroleptics, reduced to the highest degree the cortisol secretion in the first 30% of the hospitalization period. In the case of SSRIs, SNRIs, and polypragmasy, the reduction of the hormone secretion was also retained to up the end of the hospitalization. It can thus be concluded that the inhibition of the secretion is stable.

Almost all patients treated with polypragmasy are grouped in clusters Ib and II, both in the HCA dendrogram and the PCA score plot. It is known that combined treatment is only used, when a patient does not respond to the treatment with one drug. In this case the cortisol secretion is inhibited by two or even three drugs with different mechanisms of action.

HCA and PCA have also demonstrated that neuroleptics, which are also used for the treatment of depression, did not create a separate cluster. In this case, almost all the patients treated with antipsychotic drugs are grouped in cluster I. This suggests that neuroleptics affected cortisol secretion similarly as did antidepressants.

## 5. Conclusions

This study has shown that various groups of antidepressants affect in the varying degree the cortisol level. SSRIs and SNRIs, but also polypragmasy most effectively suppress the hormone secretion. The results of this study were confirmed by HCA and PCA. Both multivariate statistical techniques can be used as complementary tools for interpretation of the results obtained with the aid of laboratory diagnostic methods.

These analyses suggest that the determination of cortisol level at the beginning of the hospitalization and its decreasing during a few first days of the treatment can be helpful in prognosis of the effectiveness of therapy.

## Figures and Tables

**Table 1:** Average levels of cortisol in saliva of patients under antidepressant therapy.

Antidepressant	Active substance (dose, mg/day)	Monotherapy					Polypragmasy				
		Number of patients	Mean concentration of salivary cortisol, ng/mL				Number of patients	Mean concentration of salivary cortisol, ng/mL			
			Initial	Final	Highest	Lowest		Initial	Final	Highest	Lowest
TCAs	Amitriptyline (100 mg)	7	53.03	18.84	161.25	1.25	—				
	Clomipramine (100 mg)	3	16.46	13.08	55.00	2.50	1	72.00	5.50	72.00	2.62
	Opipramol (100 mg)	1	33.75	13.75	33.75	2.50	—				
	Doxepin (100 mg)	1	87.72	5.62	87.72	1.62	1	13.50	2.00	13.50	2.00

SSRIs	Sertraline (50 mg)	10	39.44	32.51	190.00	0.25	5	39.60	5.10	95.00	1.25
	Citalopram (40 mg)	7	59.46	6.89	93.25	0.62	3	52.42	8.42	72.00	1.75
	Escitalopram (20 mg)	5	47.46	4.77	98.50	1.25	4	39.45	5.06	61.00	1.25
	Fluvoxamine (100 mg)	3	43.54	20.75	49.75	2.50	—				
	Fluoxetine (20 mg)	1	2.50	3.75	3.75	2.50	—				
	Paroxetine (20 mg)	2	68.25	98.94	195.00	2.50	—				

SNRIs	Venlafaxine (75 mg)	14	88.32	9.13	372.5	0.25	5	41.55	5.45	95.00	2.50
SSAs	Mianserin (90 mg)	7	63.75	26.05	195.00	1.25	5	33.27	6.90	46.25	1.25
NaSSAs	Mirtazapine (45 mg)	1	33.00	8.62	33.00	6.25	1	31.25	2.75	31.25	2.50
SARIs	Trazodone (300 mg)	3	79.17	6.46	182.50	1.25	8	32.91	4.03	45.75	1.50
SSREs	Tianeptine (37.5 mg)	3	40.50	16.62	52.00	1.75	—				
RIMA	Moclobemid (600 mg)	1	37.50	5.62	37.50	1.25	—				
Others psychoactive drugs		10	44.51	12.22	133.50	1.25	1	42.50	25.00	42.50	2.50

TCAs: tricyclic antidepressants, SSRIs: selective serotonin reuptake inhibitors, SNRIs: serotonin-noradrenalin reuptake inhibitors, SSAs: specific serotonin antidepressants, NaSSAs: noradrenergic and selective serotoninergic antidepressants, SARIs: serotonin antagonist and reuptake inhibitors, SSREs: selective serotonin reuptake enhancers, and RIMA: reversible inhibitors of monoaminooxidase-A.

**Table 2:** Characteristic features of four groups of patients under antidepressant therapy displayed by HCA dendrogram.

HCA cluster	Antidepressant	Number of patients	Age of patients, years	Multiplicity of hospitalization	Hospitalization period, days	Initial and final cortisol levels, ng/mL	Highest and lowest cortisol levels, ng/mL	Mean cortisol level, ng/mL	Median for cortisol level, ng/mL
Cluster Ia	TCAs	5	51	3	29	16.57–9.67	1.25–26.25	10.80	8.44
	SSRIs	9	49	2	63	17.33–4.83	2.00–31.50	8.51	6.94
	SNRIs	2	44	1	59	15.00–5.53	1.25–24.75	8.50	7.53
	SSAs	3	39	2	66	20.58–5.21	1.25–24.25	9.30	8.75
	NaSSAs	1	63	1	21	33.00–8.62	6.25–33.00	17.50	8.62
	SSREs	1	28	7	46	27.50–9.25	1.75–27.50	8.62	7.62
	Others psychoactive drugs	4	44	3	25	12.56–7.92	1.37–21.25	7.59	6.69
	Polypragmasy	3	50	2	40	11.79–2.42	1.25–15.62	5.48	6.00

Cluster Ib	TCAs	4	55	1	71	31.06–11.59	1.25–55.00	12.98	9.15
	SSRIs	7	46	2	48	32.99–11.05	1.25–49.75	11.78	7.50
	SNRIs	5	45	2	63	33.80–12.57	1.25–62.50	9.31	4.00
	SSAs	1	57	2	46	29.50–2.87	1.25–29.50	6.34	4.62
	SARIs	2	42	5	80	27.50–7.19	1.25–37.50	9.13	7.34
	SSREs	2	47	2	21	47.00–20.31	1.75–52.00	16.24	10.69
	RIMA	1	50	5	23	37.50–5.62	1.25–37.50	10.41	6.12
	Others psychoactive drugs	4	42	3	31	34.75–7.70	1.25–38.75	11.84	6.25
	Polypragmasy	9	48	3	39	38.46–5.10	1.25–46.25	9.15	7.53

Cluster II	TCAs	2	60	1	28	86.86–6.62	1.62–87.70	22.26	10.53
	SSRIs	6	49	3	46	77.59–15.71	1.25–95.00	14.19	7.62
	SNRIs	5	45	1	38	82.25–7.85	2.50–91.00	20.97	10.00
	SSAs	2	58	1	53	58.00–6.31	1.37–102.00	12.47	7.87
	Polypragmasy	2	62	5	45	76.00–9.54	2.50–95.00	20.62	8.34

Cluster III	TCAs	1	31	3	32	161.25–83.75	8.75–161.25	50.57	30.62
	SSRIs	4	48	3	35	87.44–105.00	0.25–195.00	19.55	9.06
	SNRIs	3	44	2	51	234.96–7.72	0.25–3.75	30.03	5.94
	SSAs	1	53	6	29	195.00–151.25	5.00–195.00	26.03	11.25
	SARIs	1	51	4	76	182.50–5.00	1.25–182.50	26.73	13.00
	Others psychoactive drugs	2	45	2	33	366.27–41.25	1.25–133.50	16.97	7.28
